# Supplementary material for: The Genetic Variants Influencing Hypertension Prevalence Based on the Risk of Insulin Resistance as Assessed Using the Metabolic Score for Insulin Resistance (METS-IR)
Source: Int J Mol Sci. 2024 Nov 26;25(23):12690. doi: 10.3390/ijms252312690 (PMC11640995; doi:10.3390/ijms252312690)
Supplement: Supplementary file 1 [file ijms-25-12690-s001.zip › Supplementary Figure S1.pdf]

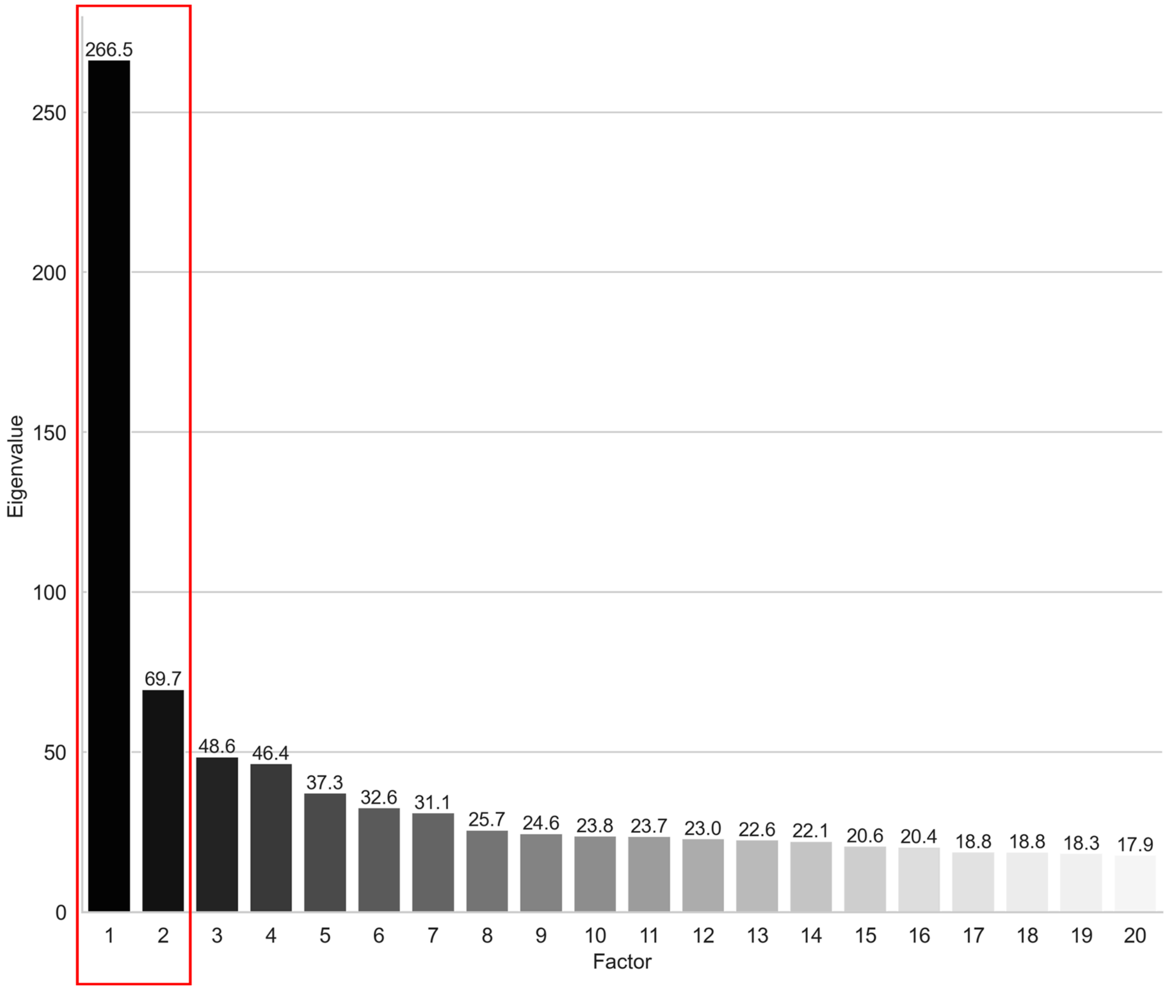

Figure S1. shows the PCA plot of study participants, demonstrating that Eigenvalue distribution is predominantly concentrated on PC1.
